# 1879. Detection of COVID-19 Outbreaks in Long-Term Care Homes Using Built Environment Testing for SARS-CoV-2: A Multicentre Prospective Study

**DOI:** 10.1093/ofid/ofac492.1506

**Published:** 2022-12-15

**Authors:** Michael Fralick, Jason Moggridge, Lucas Castellani, Sylva Donaldson, David S Guttman, Aaron Hinz, Laura Hug, Doug Manuel, Allison McGeer, Hebah Mejbel, Caroline Nott, Ashley Raudanskis, Nisha Thampi, Alex Wong, Veronica Zanichelli, Rees Kassen, Derek Macfadden

**Affiliations:** University of Toronto Department of Medicine, Toronto, Ontario, Canada; Mt Sinai Hospital, Toronto, Ontario, Canada; Sault Area Hospital, Sault Ste Marie, Ontario, Canada; University of Toronto, Toronto, Ontario, Canada; University of Toronto, Toronto, Ontario, Canada; Carleton University, Ottawa, Ontario, Canada; University of Waterloo, Waterloo, Ontario, Canada; The Ottawa Hospital Research Institute, Ottawa, Ontario, Canada; Mt. Sinai Hospital, Toronto, Ontario, Canada; Carleton University, Ottawa, Ontario, Canada; The Ottawa Hospital, Ottawa, Ontario, Canada; Mt. Sinai Hospital, Toronto, Ontario, Canada; CHEO, Ottawa, Ontario, Canada; Carleton University, Ottawa, Ontario, Canada; Ottawa Hospital Research Institute, Ottawa, Ontario, Canada; University of Ottawa, Ottawa, Ontario, Canada; Ottawa Hospital Research Institute, Ottawa, Ontario, Canada

## Abstract

**Background:**

Environmental surveillance of SARS-CoV-2 via wastewater has become an invaluable tool for population-level surveillance of COVID-19. More highly resolved environmental sampling approaches may also be useful for surveillance. Built environment sampling may provide a spatially refined approach for surveillance of COVID-19 in congregate living settings.

**Methods:**

We conducted a prospective study of 10 long-term care homes (LTCHs) in both urban and rural settings in Ontario Canada between September 2021 and April 2022. Floor surfaces were sampled weekly at multiple locations within each building and were analyzed for the presence of SARS-CoV-2 using qPCR. The exposure variable was detection of SARS-CoV-2 on floors. The primary outcome was the presence of a COVID-19 outbreak. We calculated the test characteristics of the presence of SARS-CoV-2 on floors for detection of COVID-19 outbreaks.

**Results:**

We followed 10 LTCHs for 214 cumulative weeks, and collected 3,219 swabs from 183 unique locations. Overall, 15 COVID-19 outbreaks occurred with 74.9 cumulative weeks of outbreaks. During time periods when there were outbreaks of COVID-19 the proportion of floor swabs positive for SaRS-CoV-2 was 50.8% (95% CI: 47.7-53.9). During time periods where there were no outbreaks of COVID-19 the proportion of floor swabs positive was 15.8% (95% CI:14.3-17.3). Using the proportion of positive floor swabs for SARS-CoV-2 to predict COVID-19 outbreak status for a given week, the area under the receiver operating curve was 0.84 (95% CI: 0.76-0.92). Using thresholds of ≥10%, ≥30%, and ≥50%, the prevalence of floor swabs positive for SARS-CoV-2 yielded positive predictive values for outbreak of 0.52 (0.43-0.61), 0.65 (0.53-0.75), and 0.72 (0.58-0.83) respectively, and negative predictive values of 0.93 (0.86-0.97), 0.85 (0.78-0.91), and 0.80 (0.73-0.86) respectively (Figure 1). 13 outbreaks had floor sampling performed in the week prior to them being identified, and of these 7 (54%) had positive swab proportions exceeding 30% in the week prior to the outbreak.

**Figure 1.**
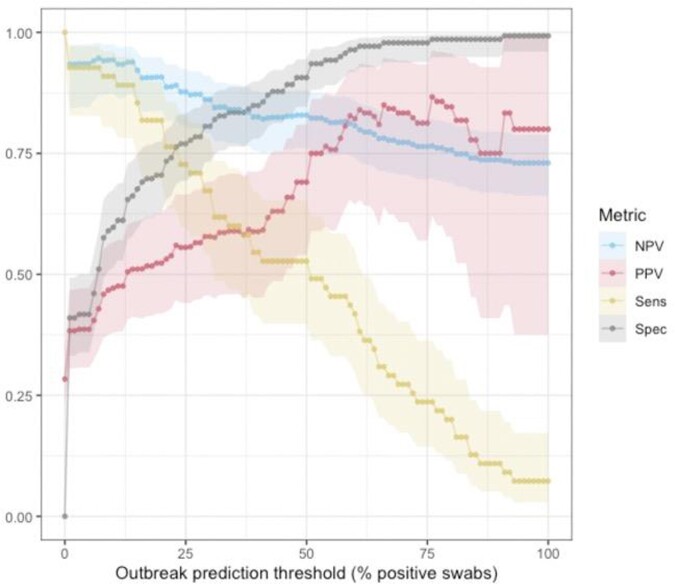
Test characteristics of built environment floor swabs for predicting COVID-19 outbreaks in LTCH.

Figure 1. Test characteristics of different thresholds for percentage of floor swabs positive for SARS-CoV-2 at a given LTCH for predicting active COVID-19 outbreak in the same building in the same week. NPV = negative predictive value, PPV = positive predictive value, Sens = sensitivity, Spec = specificity.

**Conclusion:**

Detection of SARS-CoV-2 on floors is strongly associated with COVID-19 outbreaks in LTCHs. These data suggest a potential role for floor sampling in improving early outbreak detection and management.

**Disclosures:**

**Michael Fralick, MD**, ProofDx: Advisor/Consultant **Doug Manuel, MD, PhD**, World Bank: Advisor/Consultant.

